# Dual optical elastography detects TGF-β-induced alterations in the biomechanical properties of skin scaffolds

**DOI:** 10.1117/1.JBO.29.9.095002

**Published:** 2024-09-18

**Authors:** Taye T. Mekonnen, Yogeshwari S. Ambekar, Christian Zevallos-Delgado, Achuth Nair, Fernando Zvietcovich, Hoda Zarkoob, Manmohan Singh, Yi Wei Lim, Marc Ferrer, Salavat R. Aglyamov, Giuliano Scarcelli, Min Jae Song, Kirill V. Larin

**Affiliations:** a University of Houston, Department of Biomedical Engineering, Houston, Texas, United States; b University of Sydney, Department of Mechanical Engineering, Sydney, New South Wales, Australia; c University of Maryland, Fischell Department of Bioengineering, College Park, Maryland, United States; d Pontificia Universidad Catolica del Peru, Department of Engineering, Lima, Peru; e National Institutes of Health, National Center for Advancing Translational Sciences, Rockville, Maryland, United States; f University of Houston, Department of Mechanical Engineering, Houston, Texas, United States

**Keywords:** optical coherence elastography, Brillouin microscopy, elasticity, tissue scaffold, bioengineered skin

## Abstract

**Significance:**

The skin’s mechanical properties are tightly regulated. Various pathologies can affect skin stiffness, and understanding these changes is a focus in tissue engineering. *Ex vivo* skin scaffolds are a robust platform for evaluating the effects of various genetic and molecular interactions on the skin. Transforming growth factor-beta (TGF-β) is a critical signaling molecule in the skin that can regulate the amount of collagen and elastin in the skin and, consequently, its mechanical properties.

**Aim:**

This study investigates the biomechanical properties of bio-engineered skin scaffolds, focusing on the influence of TGF-β, a signaling molecule with diverse cellular functions.

**Approach:**

The TGF-β receptor I inhibitor, galunisertib, was employed to assess the mechanical changes resulting from dysregulation of TGF-β. Skin scaffold samples, grouped into three categories (control, TGF-β-treated, and TGF-β + galunisertib-treated), were prepared in two distinct culture media—one with aprotinin (AP) and another without. Two optical elastography techniques, namely wave-based optical coherence elastography (OCE) and Brillouin microscopy, were utilized to quantify the biomechanical properties of the tissues.

**Results:**

Results showed significantly higher wave speed (with AP, p<0.001; without AP, p<0.001) and Brillouin frequency shift (with AP, p<0.001; without AP, p=0.01) in TGF-β-treated group compared with the control group. The difference in wave speed between the control and TGF-β + galunisertib with (p=0.10) and without AP (p=0.36) was not significant. Moreover, the TGF-β + galunisertib-treated group exhibited lower wave speed without and with AP and reduced Brillouin frequency shift than the TGF-β-treated group without AP, further strengthening the potential role of TGF-β in regulating the mechanical properties of the samples.

**Conclusions:**

These findings offer valuable insights into TGF-β-induced biomechanical alterations in bio-engineered skin scaffolds, highlighting the potential of OCE and Brillouin microscopy in the development of targeted therapies in conditions involving abnormal tissue remodeling and fibrosis.

## 1 Introduction

The skin features an inherent structure that provides strength and flexibility, functioning as a protective shield for softer, deeper tissues. The skin is subject to forces arising from body mechanics, such as joint movements and muscle contractions, as well as external factors, including physical insults and surgeries. Thus, the skin should be able to withstand applied forces (tensile strength), stretch without losing its structural integrity (extensibility), recoil back to its original form (elasticity), and regenerate original properties following an injury to maintain proper body functions.^1^ Nonetheless, dermal biomechanical properties may be compromised by various factors, such as aging ^2^ and disease,^3^ potentially impeding its optimal function. Therefore, understanding the alterations in the biomechanical properties of the skin is crucial in tissue engineering and regenerative medicine, particularly in designing effective skin substitutes and enhancing the success rates of skin grafts.

The mechanical properties of the skin are predominantly governed by the collagen and elastin components of the extracellular matrix (ECM), a non-cellular network of proteins and carbohydrates that provides structural and biomechanical support to the surrounding cells.^4^^,^^5^ The collagen and elastin fibers influence the tensile^6^^–^^8^ and elastic^8^^–^^11^ properties of the skin, respectively. The dysregulated composition of these fibers can disrupt tissue mechanics through excessive matrix deposition, remodeling, and degradation, potentially leading to fibrosis.^12^^–^^15^ In tissue engineering, growth factors, such as transforming growth factor-beta (TGF-β), are often used to increase the deposition of collagen and elastin in various tissues, including the skin, lungs, liver, and kidneys.^16^^,^^17^ TGF-β, a multifunctional cytokine, stimulates the synthesis of collagen and glycosaminoglycans within the skin. It influences fibroblast differentiation into myofibroblasts, playing a key role in wound contraction and remodeling.^18^ TGF-β also upregulates the expression of tissue inhibitors of metalloproteinases, which inhibit the activity of matrix metalloproteinases that are responsible for ECM degradation.^19^ This results in an imbalance between ECM synthesis and degradation, leading to the accumulation of ECM proteins and the development of fibrosis.^20^

*Ex vivo* scaffold-based skin offers a robust platform for evaluating TGF-β-induced changes in mechanical characteristics, serving as a valuable approach for developing tissue-engineered skin substitutes that mimic the structure and function of the native skin. It provides a controlled and reproducible environment for studying specific tissue responses and behavior outside the complexities of an *in vivo* setting.^21^ While there is substantial evidence elucidating the influence of TGF-β on ECM constructs,^22^^–^^24^ a notable research gap exists in exploring the mechanical properties of skin scaffolds in response to TGF-β.

Several techniques, such as tensile, suction, torsion, and twisting tests, can be used to evaluate skin scaffold mechanical properties.^25^ Nevertheless, noninvasive, *in situ* measurements are advantageous for maintaining the integrity of delicate skin scaffolds, rendering imaging techniques such as ultrasound and optical elastography more suitable due to their non-destructive nature. Research on imaging the elastic properties of the skin using ultrasound elastography (USE) is growing, such as for detecting systemic sclerosis.^26^^–^^28^ However, USE has worse structural and elastic resolution compared with optical techniques such as optical coherence elastography (OCE) and Brillouin microscopy.^29^ Prior studies have demonstrated the potential of OCE in quantifying skin biomechanical properties in both healthy^30^^–^^32^ and diseased conditions, including systemic sclerosis *in vivo* in preclinical and clinical settings.^33^^,^^34^ Wave-based OCE,^35^ air-coupled ultrasound OCE (ACUS-OCE) in particular,^36^ measures minute displacements (<10  μm) induced by non-contact acoustic radiation force (ARF). The ensuing propagation speed of the mechanical wave is then quantified, which correlates with the inherent shear modulus of the tissue.^35^ Similarly, Brillouin microscopy has shown promise for non-invasively mapping the Brillouin frequency shift (GHz range), which corresponds to the longitudinal elastic modulus (GPa range) of tissues, with high spatial resolution.^37^

In this study, we employ both ACUS-OCE and Brillouin microscopy to evaluate the biomechanical response of skin scaffolds to TGF-β treatment. Our prior work has highlighted the effectiveness of these optical techniques in offering complementary insights into tissue mechanical properties.^38^^,^^39^ A correlation may exist between the shear modulus obtained from OCE and the longitudinal modulus estimated using Brillouin microscopy due to their common dependence on tissue structural components like collagen fibers, ECM structure, and water content. However, such correlations are not always guaranteed in complex tissues, and therefore, measuring both moduli is often necessary for precise characterization, highlighting the potential of this combined approach to provide comprehensive tissue biomechanical properties. Such an approach would overcome the trade-off between the slower yet high elastic-resolution Brillouin microscopy and the faster and more quantitative imaging capabilities with a broader field of view offered by OCE.

## 2 Materials and Methods

### 2.1 Skin Scaffold Samples

Skin scaffold samples were prepared in two sets, each in a 96-well transwell plate, at the National Center for Advancing Translational Sciences (NCATS), NIH, Maryland, United States. In plate 1, aprotinin (AP) was maintained in the culture media throughout skin sample growth, whereas the AP in plate 2 was removed during the final 6 days of the culture. The removal of AP from the second group aimed to eliminate potential masking effects on stiffness differences caused by fibroblast-induced collagen secretion, as AP is known for inhibiting fibrin gel degradation.^40^ Each plate consisted of three sample subgroups, resulting in a total of six sample sets. The first subgroup served as a control (CTL), the second subgroup was treated with 10  ng/ml TGF-β, and the third subgroup was treated with both 10  ng/ml TGF-β and 10  μM of a TGF-β receptor 1 inhibitor, Galunisertib (LY2157299), hereafter referred to as galunisertib.^41^ Dimethyl sulfoxide was used as a control. The cultures were developed in transwell inserts with an effective area of $0.143\;cm^{2}$. A roughly 10-μm-thick membrane within the scaffolds separated the epidermis and dermis layers, which were cultured at the apical and basolateral sides of the insert, respectively. The samples were transported overnight to the University of Houston for elasticity imaging using Brillouin microscopy and OCE.

### 2.2 Imaging Systems and Data Acquisition

**Fig. 1:**
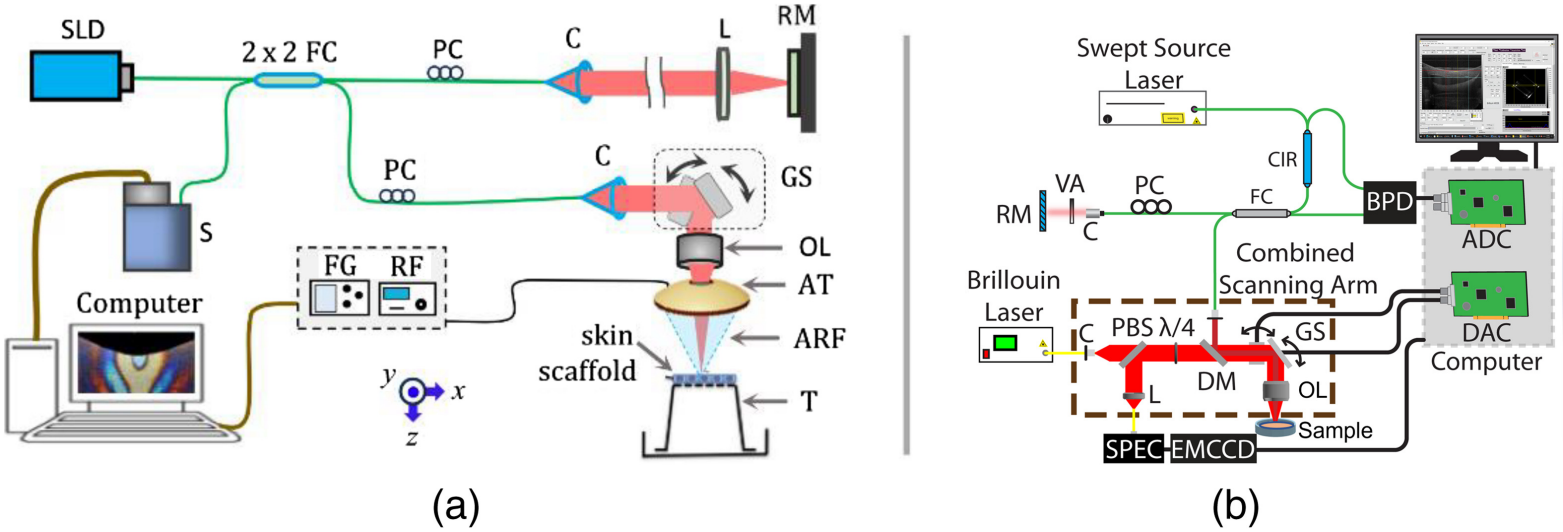
(a) Schematic of the ACUS-OCE system consisting of a hemispherical ACUS transducer to induce elastic waves in the skin scaffold and a PhS-OCT system to track the mechanical wave. The ACUS transducer had a ∼10-mm apical opening, which allows confocal OCT imaging. During imaging, the transwell was inverted with the basal side facing the imaging and excitation beams, and hence, the dermis layer was directly imaged. (b) Schematic of the Brillouin/OCT system. OCT was used to align the sample for Brillouin imaging. ARF, acoustic radiation force; AT, acoustic transducer; C, collimator; DAC, digital to analog converter; D, dichroic mirror; EMCCD, electron-multiplying CCD; FC, fiber coupler; FG, function generator; GS, 2D galvo scanner; L, lens; OL, objective lens; PBS, polarizing beam splitter; PC, polarization controller; RF, power amplifier; RM, reference mirror; S, SPEC, spectrometer; SLD, superluminescent diode; T, Transwell insert; VA, variable attenuator; VIPA, virtually imaged phase array; λ/4, quarter wave plate. MS Word 2010 Home ribbon. The red arrow indicates where to access the Styles window.

To preserve the structural integrity of the skin scaffolds and facilitate high-throughput imaging, measurements were performed directly on the intact samples within the transwell inserts. During imaging experiments, the transwell inserts were flipped to enable measurements from the basolateral side, as illustrated in Fig. 1(a). This orientation permitted the direct scanning of the dermis layer using both OCE and the Brillouin systems, thereby enabling effective assessment of the mechanical properties of the dermis. In addition, the experiments were carefully timed to ensure that measurements took place within seconds after inverting the transwell, preserving the skin structure’s freshness.

The ACUS-OCE system utilized a phase-sensitive spectral-domain OCT (PhS-OCT) system to track the propagation of low amplitude (<10  μm) mechanical waves induced by ACUS excitation. The excitation method was implemented using a hollow hemispherical ACUS transducer featuring a circular opening of 10 mm at the apex for confocal OCT imaging.^36^ Positioned at a focal distance of ∼20  mm from the sample, the ACUS transducer, operating at a 1 MHz resonant frequency, generated a focused ARF on the sample, roughly at the center of the inverted transwell insert and surface of the skin sample. To drive the transducer, a function generator (DG4162, RIGOL Tech, Beijing, China) generated a 1 MHz sinusoidal wave that was amplitude modulated with a rectangular pulse train comprising five cycles at 2 kHz. The modulated signal was then amplified using a 55-dB power amplifier (1040L, Electronics & Innovation, Ltd, Rochester, New York, United States) before driving the transducer. The induced mechanical waves were imaged using the PhS-OCT system, employing a superluminescent diode (Broadlighter S840-I-B-20, Superlum, Cobh Cross, Ireland) with a central wavelength of 840 nm and full-width-half-maximum bandwidth of ∼49  nm. The system had an axial resolution of 9  μm (in air) and a displacement sensitivity of 0.28 nm. For each sample, the data acquisition process involved two M-B mode scans,^42^ covering a lateral distance of 3.95 and 3.96 mm along the orthogonal x and y axes, respectively, as shown in Fig. 1(a). Temporal data were collected with 1000 A-lines per M-mode scan at an A-line acquisition rate of 25 kHz, and 1000 lateral locations were probed per B-scan.

A custom MATLAB^®^ R2023a script (Mathworks, Inc., Massachusetts, United States) was used to process the spatio-temporal data. Briefly, standard OCE signal processing techniques were applied to the raw M-B interferograms to remove background noise and motion artifacts.^42^^,^^43^ A spatio-temporal displacement map was generated by applying a fast Fourier transform (FFT) to the denoised data and extracting the temporally unwrapped phase at each axial pixel.^36^^,^^44^ The speed of the mechanical wave was then quantified as the slope of the linear fit of the primary peak of wave propagation to the propagation distance along the skin scaffold surface. This procedure was repeated for subsurface sample layers over a thickness of ∼250  μm, and a mean wave speed was computed for each M-B mode acquisition, enhancing the accuracy of the estimated speed.

Mechanical characterization with Brillouin microscopy was performed using a home-built multimodal Brillouin-OCT setup.^45^ The Brillouin sub-system consisted of a 660-nm single-mode laser source (Torus; Laser Quantum Inc., Fremont, California, United States), with ∼25  mW power on the sample. The Brillouin frequency shift was detected by a spectrometer composed of a dual-stage virtually imaged phase array (VIPA) spectrometer with ∼33  GHz of free spectral range. The Brillouin spectrometer was calibrated with standard materials, including water and acetone, before each measurement. Brillouin measurements were acquired with a microscope objective with 0.25 numerical aperture, resulting in a lateral resolution of ∼3.8  μm, and an axial resolution of ∼36  μm, which were measured using a beam profiler (LaserCam-HR II; Coherent Inc., Santa Clara, California, United States). The backscattered light collected from the sample was transferred to a dual-stage VIPA spectrometer through a single-mode optical fiber. An electron-multiplying charge-coupled device camera (iXon Andor, Belfast, United Kingdom) was used to detect the Brillouin frequency shift. The exposure time of the camera was 0.1 s during all measurements. Brillouin images were acquired along a 1.3-mm lateral region (10 pixels) and 0.3 mm axially (25 axial pixels along the thickness of the skin). The total acquisition period was 25 s per sample. The Brillouin system was co-aligned with a swept-source OCT system with a central wavelength of 1310 nm, bandwidth of 105 nm, and A-scan rate of 50 kHz for structural image-guided Brillouin measurements, as shown in Fig. 1(b). The multimodal system ensured the Brillouin measurements were taken at approximately the center of each transwell.

## 3 Results

**Fig. 2:**
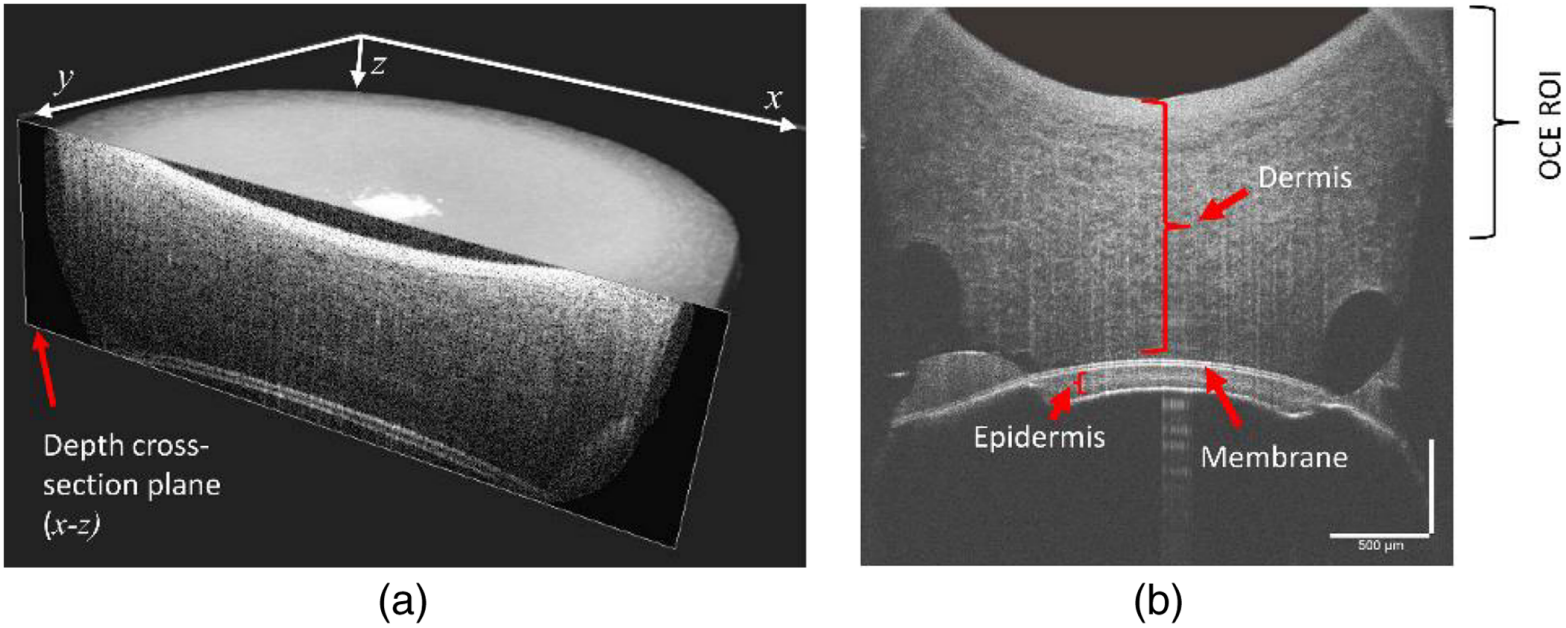
(a) 3D rendering of a typical skin scaffold sample obtained via 3D OCT scanning from the inverted (basolateral side) of the transwell. (b) Representative cross-sectional image of the skin scaffold showing various in-depth regions, including the dermis, epidermis, and membrane layers. Elastic wave speed analysis was performed in the upper portion of the dermis layer, identified as OCE ROI in the cross-sectional image.

Figure 2 shows a typical structural image of the skin scaffold reconstructed from a 3D OCT scan. OCE measurements were performed at the depth cross-sections situated in the x-z and y-z planes crossing the center of the volume image depicted in Fig. 2(a). As shown in Fig. 2(b), the structure of the tissue comprises a much thicker dermis layer at the top, a membrane layer of ∼10  μm in the middle, and an epidermis layer at the bottom. Both OCE and Brillouin imaging analyses were conducted in the dermis layer, more specifically, near the top surface of the scaffold. The structural OCT images were used for qualitative visualization of the sample integrity, and only samples where the dermis layer was still intact with the membrane were included in the data analysis to mitigate the potential effect of membrane detachment on the estimated stiffness parameters.

**Fig. 3:**
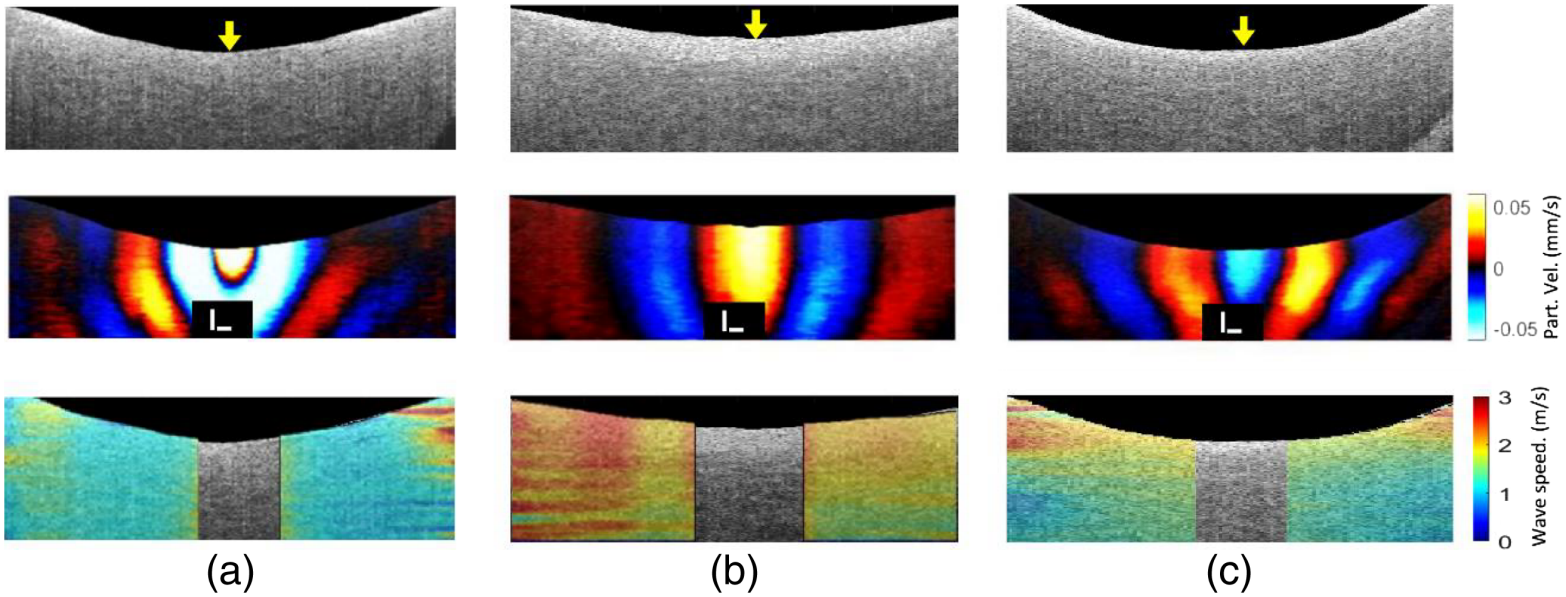
Typical B-mode images (top row), wave propagation snapshots (middle row), and speed maps (bottom row) for (a) CTL, (b) TGF-β, and (c) TGF-β + galunisertib with aprotinin (AP). ACUS excitation points are indicated by the yellow arrows. In the middle panel, the wave propagation snapshots (t=7.48  ms for CTL, t=6.08  ms for TGF-β, and t=7.16  ms for TGF-β + galunisertib after the start of excitation) show differences in the wavelengths of the waves. The bottom row depicts elastic wave speed maps overlaid on the B-mode images. The scale bar is 100 μm.

In all OCE experiments, the excitation focus point was co-aligned with the midpoint of the two orthogonal scans (i.e., B-scans in x-z and y-z planes) for each sample, as denoted by the yellow arrows in the top row of Figs. 3(a)–3(c), corresponding to CTL, TGF-β-treated, and TGF-β + galunisertib-treated skin samples, respectively. The middle row of Figs. 3(a)–3(c) displays snapshots of the wave motion, revealing noticeable differences in wavelength. The TGF-β-treated sample, shown in Fig. 3(b), exhibited a relatively longer wavelength, whereas the control sample in Fig. 3(a) had the shortest wavelength. The last row of Fig. 3 presents representative spatial speed maps superimposed on OCT structural images. This speed map illustrates a relatively higher mean wave speed for the TGF-β-treated skin scaffold compared with both the control and the TGF-β + galunisertib-treated samples. In comparison, the TGF-β-treated group exhibited a relatively higher wave speed (mean ± SD: $v_{\mathrm{AP}}=2.46\pm 0.38\;m/s$; $v_{\mathrm{noAP}}=2.64\pm 0.61\;m/s$) than both the CTL (mean ± SD: $v_{\mathrm{AP}}=1.55\pm 0.42\;m/s$; $v_{\mathrm{AP}}=1.09\pm 0.21\;m/s$) and TGF-β + galunisertib-treated (mean ± SD: $v_{\mathrm{AP}}=1.78\pm 0.32\;m/s$; $v_{\mathrm{noAP}}=1.19\pm 0.30\;m/s$) groups. However, the difference between wave speeds of TGF-β groups with and without AP was not significant (p=0.36). On the other hand, the removal of AP appeared to result in a decrease in wave speed for both the CTL and TGF-β + galunisertib-treated groups, potentially attributed to the fibrin gel degradation inhibitory effect of the AP.

**Fig. 4:**
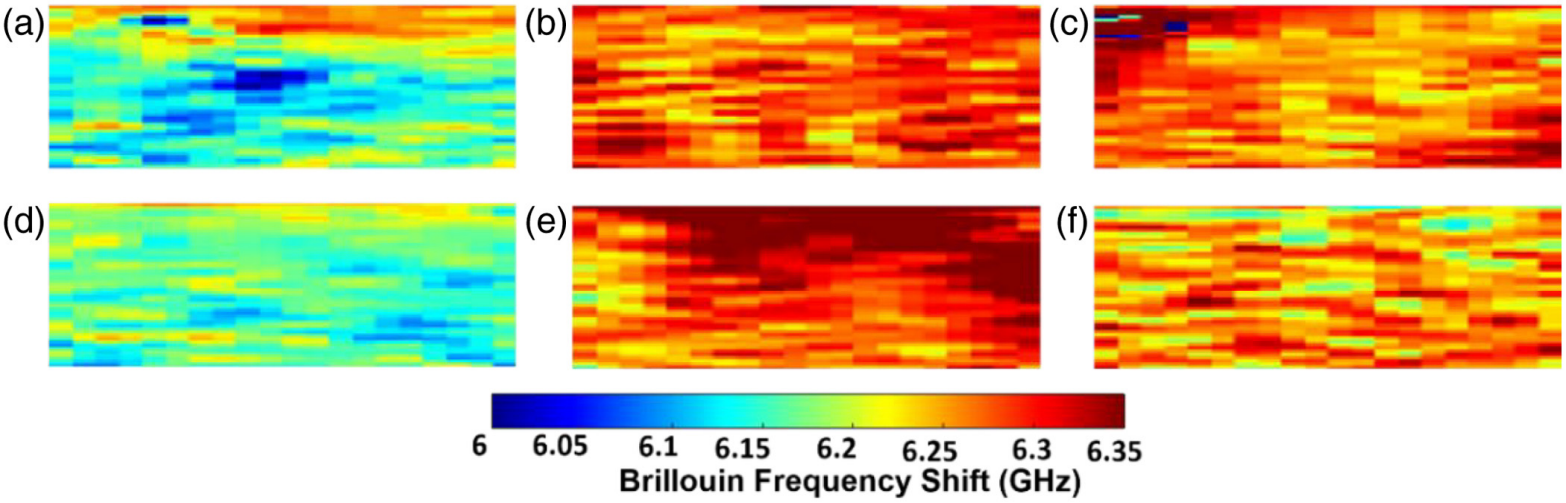
Brillouin frequency shift map of (a) CTL, (b) TGF-β, (c) TGF-β + galunisertib with AP, (d) CTL, (e) TGF-β, and (f) TGF-β + galunisertib without AP bio-engineered skin samples. Image size: 1.3  mm×0.3  mm (width × height).

Figure 4 shows typical Brillouin frequency shift maps of each treatment condition of bio-engineered skin sample, indicating a relatively lower frequency shift for the CTL groups both with and without AP as plotted in Figs. 4(a) and 4(d), respectively. The average Brillouin frequency shift of CTL, TGF-β, and TGF-β + galunisertib with AP were 6.163±0.039  GHz, 6.254±0.063  GHz, and 6.271±0.135  GHz, whereas the average Brillouin frequency shift of CTL, TGF-β, and TGF-β + galunisertib without AP were 6.171±0.021  GHz, 6.297±0.134  GHz, and 6.211±0.048  GHz.

**Fig. 5:**
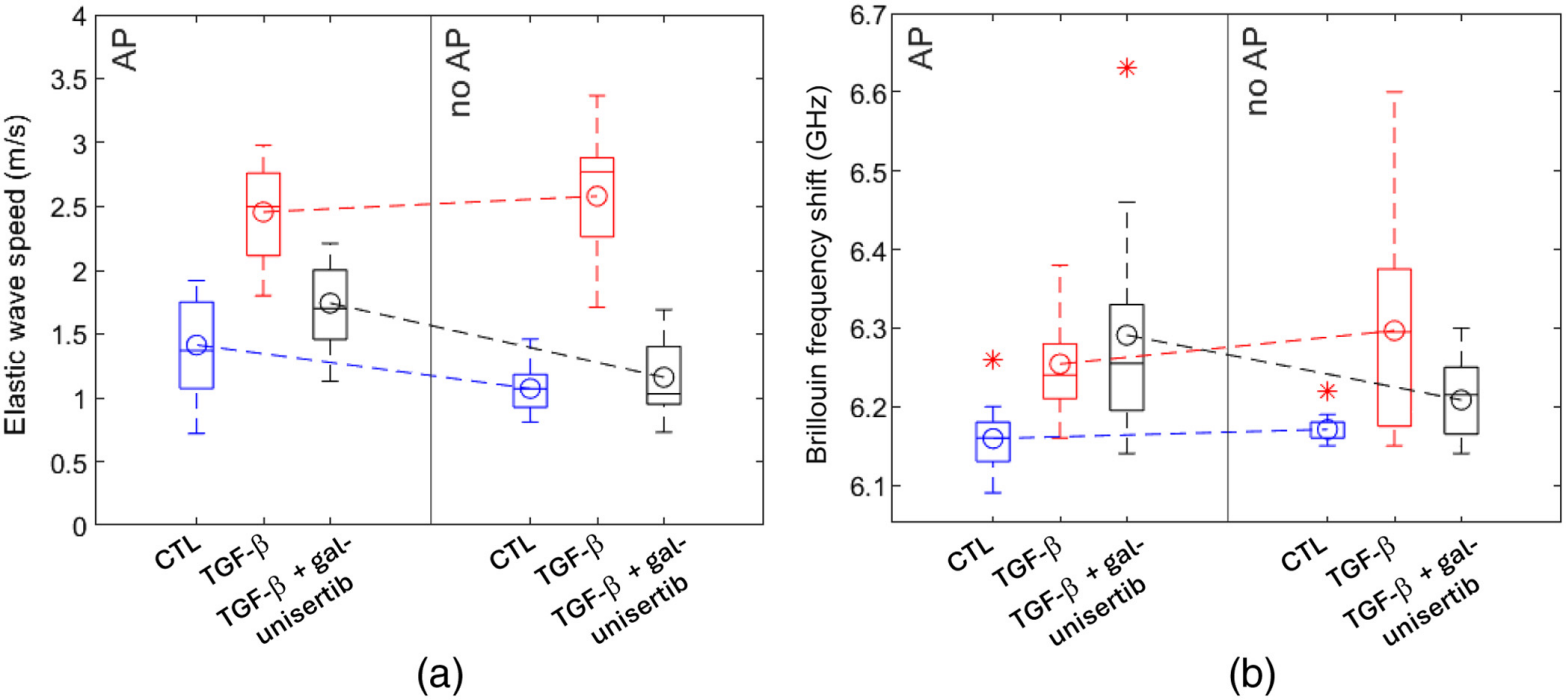
The biomechanical properties of the bio-engineered skin scaffolds, represented by (a) elastic wave speed and (b) average Brillouin frequency shift measured using OCE and Brillouin microscopy, respectively. The samples were categorized into two groups: with aprotinin (AP) and without (no AP). Outliers are indicated by *. The number of samples, N, vary between 12 and 19 among groups, as certain samples were discarded because of damage.

The box-whisker plot in Fig. 5(a) illustrates the summary of wave speed distribution for the three groups of skin scaffold samples. In the plot, the box denotes the interquartile range, the median is represented by the horizontal line within the box, and mean values are depicted by small blue circles connected with a dashed line. The whiskers extend from the minimum to maximum values of the dataset. Table 1 shows a summary of pair-wise comparisons between different groups using the Mann-Whitney statistical test. Entries below the dashed zig-zag diagonal line correspond to results for OCE, whereas those above the line pertain to Brillouin microscopy frequency shift comparison. The table entries correspond to p-values obtained by comparing the variables in the 2^nd^ row and 2^nd^ column of the corresponding entry. For example, the TGF-β-treated group with AP showed a significantly higher wave speed compared to the CTL (p<0.001) and TGF-β + galunisertib (p<0.001) groups with AP. The difference between the CTL and TGF-β + galunisertib with (p=0.10) and without AP (p=0.36) was not significant.

**Table 1:** Pair-wise statistical analysis between groups.

		AP			No AP		
		CTL	TGF-β	TGF-β + galunisertib	CTL	TGF-β	TGF-β + galunisertib
AP	CTL	—	<0.001*	0.003*	0.358	0.001*	0.006*
	TGF-β	<0.001*	—	0.762	<0.001*	0.506	0.127
	TGF-β + galunisertib	0.103	<0.001*	—	0.026*	0.500	0.345
No AP	CTL	0.001*	<0.001*	<0.001*	—	0.010*	0.015*
	TGF-β	<0.001*	0.357	0.001*	<0.001*	—	0.114
	TGF-β + galunisertib	0.012*	<0.001*	<0.001*	0.365	<0.001*	—

Brillouin results are above the dashed diagonal zig-zag, and OCE results are below.

* Indicates a statistically significant difference (P<0.05 by Mann-Whitney test).

The mean Brillouin frequency shift of every sample is plotted in the box-whisker plot in Fig. 5(b), showing the six-number boundary values of mean, median, minimum, maximum, first, and third quartiles. The trends of increasing and decreasing Brillouin frequency shift for TGF-β and TGF-β + galunisertib, respectively, agree with the changes in wave speed observed in OCE results without AP as shown in Fig. 5. However, there was no significant difference in the Brillouin frequency shift between CTL groups with and without AP (p=0.36), in contrast to the notably lower wave speed (p<0.001) for the CTL group without AP as compared to that with AP. P-values resulting from the Mann-Whitney test comparing the Brillouin frequency shift among different groups are presented above the dashed diagonal zig-zag line in Table 1. Results show that the Brillouin frequency shift of the TGF-β-treated group was significantly higher than that of CTL, both with (p<0.001) and without AP (p=0.01). Conversely, the effect of galunisertib was not statistically significant based on the comparison of Brillouin frequency shift between TGF-β and TGF-β + galunisertib, both with AP (p=0.76) and without it (p=0.11), potentially implying the inhibitor’s successful dysregulation of TGF-β.

## 4 Discussion and Conclusions

In this study, we investigated the biomechanical properties of bio-engineered skin scaffolds, specifically examining the impact of TGF-β, a signaling molecule involved in diverse cellular processes, including cell growth, differentiation, and immune response.^46^^,^^47^ Dysregulation of TGF-β signaling has been implicated in the development and progression of certain diseases, such as cancer^48^^,^^49^ and fibrosis.^50^^,^^51^ To explore this aspect, we employed galunisertib, a TGF-β receptor 1 inhibitor. Experimental investigation of evaluating the effects of both TGF-β treatment and the inhibitor involved OCE and Brillouin microscopy to quantify biomechanical parameters, specifically elastic wave speed and Brillouin frequency shift, respectively. Both the elastic wave speed and Brillouin frequency shift, which are related to the inherent mechanical properties of tissue, were quantified for the dermis layer, as shown in Fig. 2(b).

The OCE results, illustrated in Figs. 3 and 5(a) and Table 1, revealed significant differences (with AP, p<0.001; without AP, p<0.001) in wave speed between the control and TGF-β-treated groups, with higher wave speed observed in the TGF-β-treated group. Brillouin microscopy results, depicted in Figs. 4 and 5(b), complemented these findings, showing a consistent trend in Brillouin frequency shift aligned with OCE results. The increased wave speed may be attributed to TGF-β’s modulation of mechanical properties, primarily through its influence on both ECM and cellular components. For example, TGF-β promotes the synthesis of various collagen types (e.g., types I and III), leading to elevated collagen deposition, which enhances the mechanical strength of the skin.^52^ Furthermore, TGF-β influences fibroblasts, the cells involved in ECM production, triggering their conversion into highly contractile myofibroblasts.^53^ This transformation can impact the skin’s mechanical properties by influencing tension and contractility.^54^^–^^56^ Our study also showed that the TGF-β receptor inhibitor galunisertib can effectively dysregulate the influence of TGF-β as evidenced by a significantly lower wave speed (with AP, p<0.001; without AP, p<0.001) in TGF-β + galunisertib-treated skin samples as compared with the TGF-β-treated group. The Brillouin frequency shift in TGF-β was not significantly greater than that of the TGF-β + galunisertib-treated group (with AP, p=0.76; without AP, p=0.11). Similarly, the difference in the wave speed between the TGF-β + galunisertib-treated and CTL groups was also not significant (with AP, p=0.10; without AP, p=0.36). However, the comparison of Brillouin shifts between CTL and TGF-β + galunisertib showed a significantly higher frequency shift for TGF-β + galunisertib as compared with that of CTL (with AP, p=0.003; without AP, p=0.015). This disparity in results between the two techniques may be explained by their sensitivity to different mechanical properties. The Brillouin frequency shift is linked to the longitudinal modulus,^37^ whereas elastic wave speed is associated with the shear modulus,^30^^,^^35^ and these two moduli are not directly comparable. Furthermore, the complex structure and potential mechanical anisotropy of the sample, governed by collagen fiber alignment, cellular arrangement, and ECM organization, could contribute to the weak correlation in the two moduli. For instance, TGF-β inhibitor treatment may alter these structural elements of the scaffold, leading to varying impacts on longitudinal and shear moduli. Future research may explore how TGF-β inhibitor treatment alters the scaffold’s structural elements and their relationship with the two mechanical moduli, making the proposed dual elastography approach essential for detailed and comprehensive biomechanical characterization.

Differences in elastic wave speed and Brillouin frequency shift were observed between samples with and without the protease inhibitor (AP), potentially due to its effects on fibrin gel properties.^40^ Both CTL and TGF-β + galunisertib samples exhibited a decreasing tendency in wave speed without AP. Similarly, Brillouin frequency shift was lower in TGF-B + galunisertib samples without AP than with AP, with no significant difference observed in CTL samples. These changes in wave speed and Brillouin frequency shift with AP may be attributed to AP’s role in maintaining function and controlling fibrin gel degradation.^15^ On the other hand, TGF-β-treated samples (untreated with inhibitor) demonstrated a relative increase in both wave speed and Brillouin frequency shift, highlighting its impact on increasing scaffold stiffness. Generally, the results of the current study are in good agreement with previous research, which indicated a positive correlation between the tensile strength of skin and TGF-β.^57^

This study provides valuable insights into the biomechanical changes induced by TGF-β treatment and its inhibition in bio-engineered skin scaffolds. ACUS-OCE enables the noninvasive measurement of mechanical wave speed in near real-time. Physical tissue compliance descriptors, including shear modulus and viscosity, can be derived from wave propagation using appropriate elastic wave models that account for the thickness of the dermis layer and its boundary conditions.^35^ On the other hand, Brillouin microscopy offers a microscale and depth-resolved stiffness map derived from the Brillouin frequency shift, which correlates with the longitudinal modulus.^37^^,^^38^ OCE and Brillouin microscopy offer complementary insights by probing different frequency ranges of mechanical properties, high-frequency microscopic details from Brillouin, and low-frequency macroscopic properties from OCE, enabling a thorough multi-scale assessment of skin scaffold properties. Integrating both techniques confers a vital advantage for a comprehensive understanding of the role of TGF-β in skin mechanics, thereby aiding in the formulation of targeted therapies for conditions involving aberrant tissue remodeling and fibrosis. Longitudinal studies and *in vivo* experiments can further validate and extend our findings, potentially advancing the translation of these techniques into clinical applications. Here, it is important to note that in the current study, the skin scaffold was inverted with the dermis facing the imaging optics and exposed to air, unlike *in vivo* where it is sandwiched between the epidermis and deeper tissues. The different acoustic impedance of air compared with tissue may affect the OCE wave speed measurements. Therefore, accurate estimation of mechanical properties, such as shear modulus, requires an appropriate analytical model that accounts for the dermis boundary conditions.

## Data Availability

The data utilized in this study can be shared upon request.

## References

[1] Hussain S. H. Limthongkul B. Humphreys T. R., “The biomechanical properties of the skin,” Dermatol. Surg. 39(2), 193–203 (2013). 10.1111/dsu.12095 23350638

[2] Langton A. K. et al., “Ageing significantly impacts the biomechanical function and structural composition of skin,” Exp. Dermatol. 28(8), 981–984 (2019). EXDEEY 0906-6705 10.1111/exd.13980 31152614 PMC6851988

[3] Dobrev H. P., “In vivo study of skin mechanical properties in patients with systemic sclerosis,” J. Am. Acad. Dermatol. 40(3), 436–442 (1999). JAADDB 0190-9622 10.1016/S0190-9622(99)70494-9 10071315

[4] Zhong S. P. Zhang Y. Z. Lim C. T., “Tissue scaffolds for skin wound healing and dermal reconstruction,” Wiley Interdiscip. Rev. Nanomed. Nanobiotechnol. 2(5), 510–525 (2010). 10.1002/wnan.100 20607703

[5] Bottcher-Haberzeth S. Biedermann T. Reichmann E., “Tissue engineering of skin,” Burns 36(4), 450–460 (2010). BURND8 0305-4179 10.1016/j.burns.2009.08.016 20022702

[6] Grouf J. L. et al., “Differential effects of EGF and TGF-beta1 on fibroblast activity in fibrin-based tissue equivalents,” Tissue Eng. 13(4), 799–807 (2007).1937-3341 10.1089/ten.2006.0206 17346099

[r7] Neidert M. R. et al., “Enhanced fibrin remodeling in vitro with TGF-beta1, insulin and plasmin for improved tissue-equivalents,” Biomaterials 23(17), 3717–3731 (2002). BIMADU 0142-9612 10.1016/S0142-9612(02)00106-0 12109697

[8] Oxlund H. Andreassen T. T., “The roles of hyaluronic acid, collagen and elastin in the mechanical properties of connective tissues,” J. Anat. 131(Pt 4), 611–620 (1980). JOANAY 0021-8782 7216901 PMC1233214

[r9] Ross J. J. Tranquillo R. T., “ECM gene expression correlates with in vitro tissue growth and development in fibrin gel remodeled by neonatal smooth muscle cells,” Matrix Biol. 22(6), 477–490 (2003). MTBOEC 0945-053X 10.1016/S0945-053X(03)00078-7 14667840

[r10] Kucich U. et al., “Transforming growth factor-beta stabilizes elastin mRNA by a pathway requiring active Smads, protein kinase c-delta, and p38,” Am. J. Respir. Cell Mol. Biol. 26(2), 183–188 (2002). AJRBEL 1044-1549 10.1165/ajrcmb.26.2.4666 11804868

[11] Oxlund H. Manschot J. Viidik A., “The role of elastin in the mechanical properties of skin,” J. Biomech. 21(3), 213–218 (1988). JBMCB5 0021-9290 10.1016/0021-9290(88)90172-8 3379082

[12] Aires A. et al., “Engineering multifunctional metal/protein hybrid nanomaterials as tools for therapeutic intervention and high-sensitivity detection,” Chem. Sci. 12(7), 2480–2487 (2020).1478-6524 10.1039/D0SC05215A 34164014 PMC8179251

[r13] Davidson M. D. Burdick J. A. Wells R. G., “Engineered biomaterial platforms to study fibrosis,” Adv. Healthc. Mater. 9(8), e1901682 (2020). 10.1002/adhm.201901682 32181987 PMC7274888

[r14] Porras A. M. et al., “Engineering approaches to study fibrosis in 3-D in vitro systems,” Curr. Opin. Biotechnol. 40 24–30 (2016). CUOBE3 0958-1669 10.1016/j.copbio.2016.02.006 26926460 PMC4975659

[15] Hinz B., “Tissue stiffness, latent TGF-beta1 activation, and mechanical signal transduction: Implications for the pathogenesis and treatment of fibrosis,” Curr. Rheumatol. Rep. 11(2), 120–126 (2009). 10.1007/s11926-009-0017-1 19296884

[16] Quan T. et al., “Solar ultraviolet irradiation reduces collagen in photoaged human skin by blocking transforming growth factor-beta type II receptor/Smad signaling,” Am. J. Pathol. 165(3), 741–751 (2004). AJPAA4 0002-9440 10.1016/S0002-9440(10)63337-8 15331399 PMC1618600

[17] Wu L. et al., “Transforming growth factor-beta1 fails to stimulate wound healing and impairs its signal transduction in an aged ischemic ulcer model: importance of oxygen and age,” Am. J. Pathol. 154(1), 301–309 (1999). AJPAA4 0002-9440 10.1016/S0002-9440(10)65276-5 9916944 PMC1853440

[18] Do N. N. Eming S. A., “Skin fibrosis: models and mechanisms,” Curr. Res. Transl. Med. 64(4), 185–193 (2016). 10.1016/j.retram.2016.06.003 27939457

[19] Ke Y. Wang X. J., “TGFβ signaling in photoaging and UV-induced skin cancer,” J. Invest. Dermatol. 141(4S), 1104–1110 (2021). JIDEAE 0022-202X 10.1016/j.jid.2020.11.007 33358021 PMC7987776

[20] Padmanabhan J. et al., “In vivo models for the study of fibrosis,” Adv. Wound. Care 8(12), 645–654 (2019). 10.1089/wound.2018.0909 PMC6904938 31827979

[21] Dhandayuthapani B. et al., “Polymeric scaffolds in tissue engineering application: a review,” Int. J. Polym. Sci. 2011, 19 (2011). 10.1155/2011/290602

[22] Toh W. S. Lee E. H. Cao T., “Potential of human embryonic stem cells in cartilage tissue engineering and regenerative medicine,” Stem Cell Rev. Rep. 7(3), 544–559 (2011). 10.1007/s12015-010-9222-6 21188652

[r23] Seif-Naraghi S. B. et al., “Injectable extracellular matrix derived hydrogel provides a platform for enhanced retention and delivery of a heparin-binding growth factor,” Acta Biomater. 8(10), 3695–3703 (2012). 10.1016/j.actbio.2012.06.030 22750737 PMC3429632

[24] Lee H. P. et al., “Mechanical confinement regulates cartilage matrix formation by chondrocytes,” Nat. Mater. 16(12), 1243–1251 (2017). NMAACR 1476-1122 10.1038/nmat4993 28967913 PMC5701824

[25] Agache P. Humbert P., Measuring the skin, Springer Science & Business Media, Berlin (2004).

[26] Kang T. et al., “Skin imaging in systemic sclerosis,” Eur. J. Rheumatol. 1(3), 111–116 (2014). EJRIDH 0140-1610 10.5152/eurjrheumatol.2014.036 27708890 PMC5042219

[r27] Liu H. et al., “A preliminary study of skin ultrasound in diffuse cutaneous systemic sclerosis: does skin echogenicity matter?” PLoS One 12(3), e0174481 (2017). POLNCL 1932-6203 10.1371/journal.pone.0174481 28339492 PMC5365121

[28] Iagnocco A. et al., “Ultrasound elastography assessment of skin involvement in systemic sclerosis: lights and shadows,” J. Rheumatol. 37(8), 1688–1691 (2010). JORHE3 10.3899/jrheum.090974 20551100

[29] Kennedy B. F. Wijesinghe P. Sampson D. D., “The emergence of optical elastography in biomedicine,” Nat. Photonics 11(4), 215–221 (2017). NPAHBY 1749-4885 10.1038/nphoton.2017.6

[30] Liang X. Boppart S. A., “Biomechanical properties of in vivo human skin from dynamic optical coherence elastography,” IEEE Trans. Biomed. Eng. 57(4), 953–959 (2010). IEBEAX 0018-9294 10.1109/TBME.2009.2033464 19822464 PMC3699319

[r31] Feng X. et al., “In vivo stiffness measurement of epidermis, dermis, and hypodermis using broadband Rayleigh-wave optical coherence elastography,” Acta Biomater. 146, 295–305 (2022). 10.1016/j.actbio.2022.04.030 35470076 PMC11878153

[32] Bartolini L. et al., “Toward clinical elastography of dermal tissues: a medical device to probe skin’s elasticity through suction, with subsurface imaging via optical coherence tomography,” Rev. Sci. Instrum. 91(7), 074101 (2020). RSINAK 0034-6748 10.1063/5.0009639 32752846

[33] Du Y. et al., “Rapid, noninvasive quantitation of skin disease in systemic sclerosis using optical coherence elastography,” J. Biomed. Opt. 21(4), 046002 (2016). JBOPFO 1083-3668 10.1117/1.JBO.21.4.046002 27048877 PMC4837197

[34] Liu C. H. et al., “Translational optical coherence elastography for assessment of systemic sclerosis,” J. Biophotonics 12(12), e201900236 (2019). 10.1002/jbio.201900236 31343837 PMC7184265

[35] Zvietcovich F. Larin K. V., “Wave-based optical coherence elastography: the 10-year perspective,” Prog. Biomed. Eng. 4(1), 012007 (2022). 10.1088/2516-1091/ac4512 PMC8856668 35187403

[36] Zvietcovich F. et al., “Confocal air-coupled ultrasonic optical coherence elastography probe for quantitative biomechanics,” Opt. Lett. 45(23), 6567–6570 (2020). OPLEDP 0146-9592 10.1364/OL.410593 33258863 PMC10041740

[37] Prevedel R. et al., “Brillouin microscopy: an emerging tool for mechanobiology,” Nat. Methods 16(10), 969–977 (2019).1548-7091 10.1038/s41592-019-0543-3 31548707

[38] Ambekar Y. S. et al., “Multimodal quantitative optical elastography of the crystalline lens with optical coherence elastography and Brillouin microscopy,” Biomed. Opt. Express 11(4), 2041–2051 (2020). BOEICL 2156-7085 10.1364/BOE.387361 32341865 PMC7173892

[39] Nair A. et al., “Multimodal heartbeat and compression optical coherence elastography for mapping corneal biomechanics,” Front. Med. 9, 833597 (2022). 10.3389/fmed.2022.833597 PMC9037093 35479957

[40] Janmey P. A. Winer J. P. Weisel J. W., “Fibrin gels and their clinical and bioengineering applications,” J. R. Soc. Interface 6(30), 1–10 (2009).1742-5689 10.1098/rsif.2008.0327 18801715 PMC2575398

[41] Yingling J. M. et al., “Preclinical assessment of galunisertib (LY2157299 monohydrate), a first-in-class transforming growth factor-beta receptor type I inhibitor,” Oncotarget 9(6), 6659–6677 (2018). 10.18632/oncotarget.23795 29467918 PMC5805504

[42] Singh M. Zvietcovich F. Larin K. V., “Introduction to optical coherence elastography: tutorial,” J. Opt. Soc. Am. A Opt. Image Sci. Vis. 39(3), 418–430 (2022). 10.1364/JOSAA.444808 35297425 PMC10052825

[43] Song S. Huang Z. Wang R. K., “Tracking mechanical wave propagation within tissue using phase-sensitive optical coherence tomography: motion artifact and its compensation,” J. Biomed. Opt. 18(12), 121505 (2013). JBOPFO 1083-3668 10.1117/1.JBO.18.12.121505 24150274

[44] Kirby M. A. et al., “Optical coherence elastography in ophthalmology,” J. Biomed. Opt. 22(12), 121720 (2017). JBOPFO 1083-3668 10.1117/1.JBO.22.12.121720 29275544 PMC5745712

[45] Ambekar Y. S. et al., “Multimodal imaging system combining optical coherence tomography and Brillouin microscopy for neural tube imaging,” Opt. Lett. 47(6), 1347–1350 (2022). OPLEDP 0146-9592 10.1364/OL.453996 35290310 PMC9088521

[46] Moustakas A. et al., “Mechanisms of TGF-beta signaling in regulation of cell growth and differentiation,” Immunol. Lett. 82(1–2), 85–91 (2002). IMLED6 0165-2478 10.1016/S0165-2478(02)00023-8 12008039

[47] Letterio J. J. Roberts A. B., “Regulation of immune responses by TGF-beta,” Annu. Rev. Immunol. 16 137–161 (1998). ARIMDU 0732-0582 10.1146/annurev.immunol.16.1.137 9597127

[48] Syed V., “TGF-beta signaling in cancer,” J. Cell Biochem. 117(6), 1279–1287 (2016). 10.1002/jcb.25496 26774024

[49] Massague J. Sheppard D., “TGF-beta signaling in health and disease,” Cell 186(19), 4007–4037 (2023). CELLB5 0092-8674 10.1016/j.cell.2023.07.036 37714133 PMC10772989

[50] Raaf L. et al., “Myocardial fibrosis and TGFB expression in hyperhomocysteinemic rats,” Mol. Cell Biochem. 347(1–2), 63–70 (2011). 10.1007/s11010-010-0612-5 20938722

[51] Van Laethem J. L. et al., “Transforming growth factor beta promotes development of fibrosis after repeated courses of acute pancreatitis in mice,” Gastroenterology 110(2), 576–582 (1996). GASTAB 0016-5085 10.1053/gast.1996.v110.pm8566606 8566606

[52] Reddy G. K. Enwemeka C. S., “A simplified method for the analysis of hydroxyproline in biological tissues,” Clin. Biochem. 29 (3), 225–229 (1996). 10.1016/0009-9120(96)00003-6 8740508

[53] Hinz B., “Formation and function of the myofibroblast during tissue repair,” J. Invest. Dermatol. 127(3), 526–537 (2007). JIDEAE 0022-202X 10.1038/sj.jid.5700613 17299435

[54] Hinz B. et al., “The myofibroblast: one function, multiple origins,” Am. J. Pathol. 170(6), 1807–1816 (2007). AJPAA4 0002-9440 10.2353/ajpath.2007.070112 17525249 PMC1899462

[r55] Wipff P. J. et al., “Myofibroblast contraction activates latent TGF-beta1 from the extracellular matrix,” J. Cell Biol. 179(6), 1311–1323 (2007). JCLBA3 0021-9525 10.1083/jcb.200704042 18086923 PMC2140013

[56] Hinz B., “Myofibroblasts,” Exp. Eye Res. 142, 56–70 (2016). EXERA6 0014-4835 10.1016/j.exer.2015.07.009 26192991

[57] Widodo A. Rahajoe P. S. Astuti R. T., “TGF-beta expression and wound tensile strength after simple interrupted suturing and zip surgical skin closure (*in vivo* study),” Ann. Med. Surg. 58, 187–193 (2020). 10.1016/j.amsu.2020.08.009 PMC7505902 32994982

